# Prognostic value of the systemic immune-inflammation index in bladder cancer: an update evidence-based analysis

**DOI:** 10.3389/fonc.2025.1707657

**Published:** 2025-10-24

**Authors:** Jingxing Bai, Yin Huang, Bo Chen, Biao Ran, Shibo Jian, Jinze Li, Jie Chen, Qian Wei, Dehong Cao, Liangren Liu

**Affiliations:** ^1^ Department of Urology/Institute of Urology, West China Hospital, Sichuan University, Chengdu, Sichuan, China; ^2^ Department of Urology, People’s Hospital of Deyang City, Chengdu University of Traditional Chinese Medicine, Deyang, China

**Keywords:** bladder cancer, systemic immune-inflammation index, prognostic indicators, overall survival, recurrence-free survival

## Abstract

**Background:**

Bladder cancer (BC) prognosis remains challenging to predict accurately with conventional tools. Systemic immune-inflammation index (SII) has emerged as a promising biomarker reflecting the tumor microenvironment. However, existing studies are limited by small sample sizes, heterogeneous designs, and inconsistent endpoints. This updated meta-analysis aims to comprehensively evaluate the association between high SII and key survival outcomes in BC patients.

**Methods:**

We systematically searched PubMed, Embase, Web of Science, and Cochrane up to August 2025. Cohort studies reporting hazard ratios (HRs) for overall survival (OS), recurrence-free survival (RFS), progression-free survival (PFS), or cancer-specific survival (CSS) comparing high vs. low SII groups in histologically confirmed BC were included. Study quality was assessed using the Newcastle-Ottawa Scale. Pooled HRs with 95% confidence intervals (CIs) were calculated using a random-effects model. Subgroup analyses by pathological type (NMIBC vs. MIBC) and sensitivity analyses were performed. Publication bias was evaluated via funnel plots and Egger’s test.

**Results:**

Sixteen cohort studies involving 2,352 patients were analyzed. Meta-analysis revealed that elevated SII was significantly associated with worse OS (HR=1.66, 95% CI: 1.30–2.12, P < 0.0001) and RFS (HR=1.50, 95% CI: 1.28–1.76, P < 0.00001), with substantial heterogeneity (OS: I² = 81%; RFS: I² = 59%). Subgroup analysis showed significant predictive value of SII for RFS in both NMIBC (HR=1.55, 95% CI: 1.27–1.89, P < 0.0001; heterogeneity reduced to I² = 37%) and MIBC (HR=1.13, 95% CI: 1.01–1.26, P=0.03). However, OS subgroup associations for NMIBC (HR=1.15, P=0.50) and MIBC (HR=1.92, P=0.07) were non-significant. No significant associations were found for PFS (HR=1.55, 95% CI: 0.92–2.60, P=0.10, I² = 68%) or CSS (HR=1.50, 95% CI: 0.95–2.37, P=0.08, I² = 69%), likely due to limited study numbers (4 and 3, respectively). Significant publication bias was detected for OS and RFS.

**Conclusion:**

Elevated SII is significantly associated with poorer overall and recurrence-free survival in bladder cancer patients, particularly highlighting its potential predictive value for recurrence risk in NMIBC. However, significant heterogeneity, publication bias, and retrospective design limitations necessitate caution in interpretation. Future large-scale, prospective studies with standardized SII measurement and dynamic monitoring are crucial to validate its clinical utility and define optimal cut-offs for integration into risk-stratified management strategies.

**Systematic review registration:**

https://www.crd.york.ac.uk/PROSPERO/view/CRD420251145769, Prospero identifier, CRD420251145769.

## Introduction

Bladder cancer is the tenth most common malignancy worldwide, and its burden continues to grow (1). In 2020, an estimated 573,000 people were diagnosed with bladder cancer worldwide, equivalent to 5.6 cases per 100,000 people (2). Clinically, non-muscle-invasive bladder cancer (NMIBC) and muscle-invasive bladder cancer (MIBC) differ fundamentally in their biological behavior and treatment strategies. While NMIBC patients enjoy a five-year survival rate exceeding 85%, recurrence rates are as high as 60%-70% (3, 4). Meanwhile, even after radical cystectomy, 50% of MIBC patients will develop metastases within two years (5). Current prognostic assessment relies primarily on traditional indicators such as TNM staging and histological grade, but these static parameters are unable to dynamically reflect the evolving tumor microenvironment. In particular, for patients with intermediate- and high-risk NMIBC undergoing transurethral resection of bladder tumors (TURBT), existing prognostic tools, such as the European Organization for Research and Treatment (EORTC) scoring system, misjudge recurrence risk by over 30% (6). This clinical dilemma has driven the exploration of novel biomarkers, with systemic inflammation, a key area of ​​research, becoming a hot topic due to its central role in tumor progression. Tumor-associated inflammation creates a microenvironment conducive to tumor growth and metastasis by promoting angiogenesis, accelerating DNA damage, and suppressing anti-tumor immunity (7). Basic research has demonstrated that interleukin-6 (IL-6) and tumor necrosis factor-α (TNF-α) released by neutrophils can activate the STAT3 pathway to induce bladder cancer cell proliferation (8, 9), while platelet-derived microparticles promote epithelial-mesenchymal transition through transforming growth factor-β (TGF-β) signaling (10). These findings provide a theoretical foundation for the application of inflammatory markers in the prognostic assessment of bladder cancer.

The Systemic Inflammatory Index (SII), an emerging comprehensive inflammatory marker, integrates neutrophil, platelet, and lymphocyte counts (calculated as: SII = neutrophil count × platelet count/lymphocyte count), providing a multidimensional quantification of systemic inflammatory status (11, 12). Compared to single indices such as the neutrophil-lymphocyte ratio (NLR) or platelet-lymphocyte ratio (PLR), the SII provides a more comprehensive quantification of the systemic inflammatory state and the balance of pro-tumor and anti-tumor immunity in the tumor microenvironment by integrating neutrophil, platelet and lymphocyte counts into a comprehensive indicator, which may give it a superior prognostic prediction potential (13, 14). In the field of bladder cancer, the SII is particularly valuable in pathophysiology: surgical stress or the tumor itself can induce the release of immature neutrophils from the bone marrow (15). The arginase 1 (ARG1) secreted by these neutrophils depletes arginine in the microenvironment, directly inhibiting CD8+ T cell function (16). Activated platelets, through P-selectin, mediate tumor cell-endothelial cell adhesion, promoting hematogenous metastasis (17). A retrospective cohort study by Salari et al. demonstrated that elevated SII was significantly associated with worse OS, highlighting its potential as a marker of disease aggressiveness (18). However, existing evidence is conflicting: a retrospective study by Demirci et al. of 173 elderly patients with NMIBC demonstrated no significant association between SII and OS (19). This discrepancy may be due to methodological heterogeneity, including differences in the timing of SII testing, cutoff definition, and endpoint criteria.

Although more than a dozen studies have explored the value of SII in bladder cancer prognosis, a systematic evidence synthesis is lacking. Previous studies have three key limitations: first, the sample size is generally insufficient, resulting in weak statistical power; second, the study designs are highly heterogeneous, with patients included at different stages of treatment, such as after TURBT surgery, before radical cystectomy, and with BCG maintenance therapy, and the confounding effects of adjuvant therapy are not adequately controlled; finally, the definitions of prognostic endpoints are inconsistent, including multiple indicators such as overall survival (OS), recurrence-free survival (RFS), and progression-free survival (PFS), making data comparability difficult. This study, the latest and largest comprehensive evidence-based evaluation of the prognostic value of SII in bladder cancer, strictly adheres to the PRISMA 2020 statement. We systematically searched four major databases (PubMed/Embase/Web of Science/Cochrane) up to August 2025. Using a random-effects model, we integrated data from 16 cohort studies totaling 2,352 patients. We focused on analyzing the strength of the association between SII and key survival outcomes (OS, RFS, PFS, and CSS). We also explored the robustness of our results through pathological subgroup analyses and sensitivity testing, ultimately providing evidence-based decision-making for clinical practice.

## Methods

### Literature search

This meta-analysis was performed according to the PRISMA (Preferred Reporting Items for Systematic Reviews and Meta-Analysis) 2020 statement (20) and has been prospectively registered in the PROSPERO (CRD420251145769). The PRISMA checklist was presented in **Supplementary Table S1**. We conducted a systematic literature search via PubMed, Embase, Web of Science, Cochrane up to August, 2025 for studies that evaluated the role of SII in the prognosis of patients with bladder cancer. We searched the literature through the following terms: “Urinary Bladder Neoplasms”, “systemic immune-inflammation index”, and “SII”. The detailed search strategies in PubMed are as follows: ((systemic immune-inflammation index) OR (SII)) AND ((“Urinary Bladder Neoplasms”[Mesh]) OR ((((((((Urinary Bladder Neoplasm) OR (Bladder Neoplasm)) OR (Bladder Tumor)) OR (Urinary Bladder Cancer)) OR (Bladder Cancer)) OR (Cancer of Bladder)) OR (Malignant Tumor of Urinary Bladder)) OR (Bladder Carcinoma))). Furthermore, we manually screened the bibliography lists of all included studies. Two authors retrieved and assessed eligible articles independently. Any differences in literature retrieval were resolved by discussion. Searching details were shown in **Supplementary Table S2**.

### Inclusion and exclusion criteria

Articles were eligible when meeting the following standards:

P: Patients diagnosed with bladder cancer by pathology.
E: Patients with high SII levels (high and low criteria depend on the cut-off of each study).
C: Patients with low SII levels (high and low criteria depend on the cut-off of each study).
O: at least one survival outcome (OS, PFS, RFS, CSS, etc.) was evaluated.
S: study design was randomized controlled trial, cohort, or case-control.

We excluded study protocols, unpublished studies, non-original studies (including letters, comments, abstracts, correction, and reply), studies without sufficient data, and reviews.

### Data abstraction

Data abstraction was conducted by two authors severally. Any differences were settled by another author. We abstracted following information from eligible studies: first author name, published year, study region, study design, research population, sample size, age, gender, TNM stage, SII cut-off, OS, RFS, PFS, CSS. If the research data is insufficient, corresponding authors were contacted for full data if available.

### Quality evaluation

The Newcastle-Ottawa Scale (NOS) was applied for assessing the quality of included cohort studies (21), and studies with 7–9 points were considered as high quality (22). Studies with NOS scores below 6 were not included for quantitative analysis. Two authors severally assessed the quality of all included studies, and any disagreement was settled by discussion.

### Statistical analysis

Meta-analysis was conducted in Review Manager 5.4.1 edition. HR were used for the synthesis of survival data. Metrics were presented with 95%CIs. The chi-squared (χ^2^) test (Cochran’s *Q*) and inconsistency index (*I* ^2^) were applied for the evaluation of the heterogeneity of each outcome (23). χ^2^ *P* value less than 0.1 or *I* ^2^ more than 50% were regarded as high heterogeneity. The random-effects model was applied to calculate the total HR for each outcome. In addition, we conducted sensitivity analysis to assess the influence of every included study on the total HR. To identify the source of heterogeneity, we performed subgroup analysis according to the pathological type of bladder cancer. Moreover, we assessed the potential publication bias by producing funnel plots through Review Manager 5.4.1 edition as well as through performing Egger’s regression tests (24) through Stata 15.1 edition (Stata Corp, College Station, Texas, USA). *P* value < 0.05 was considered as statistically significant publication bias.

## Results

### Literature retrieval, study characteristics, and baseline

**Figure 1:**
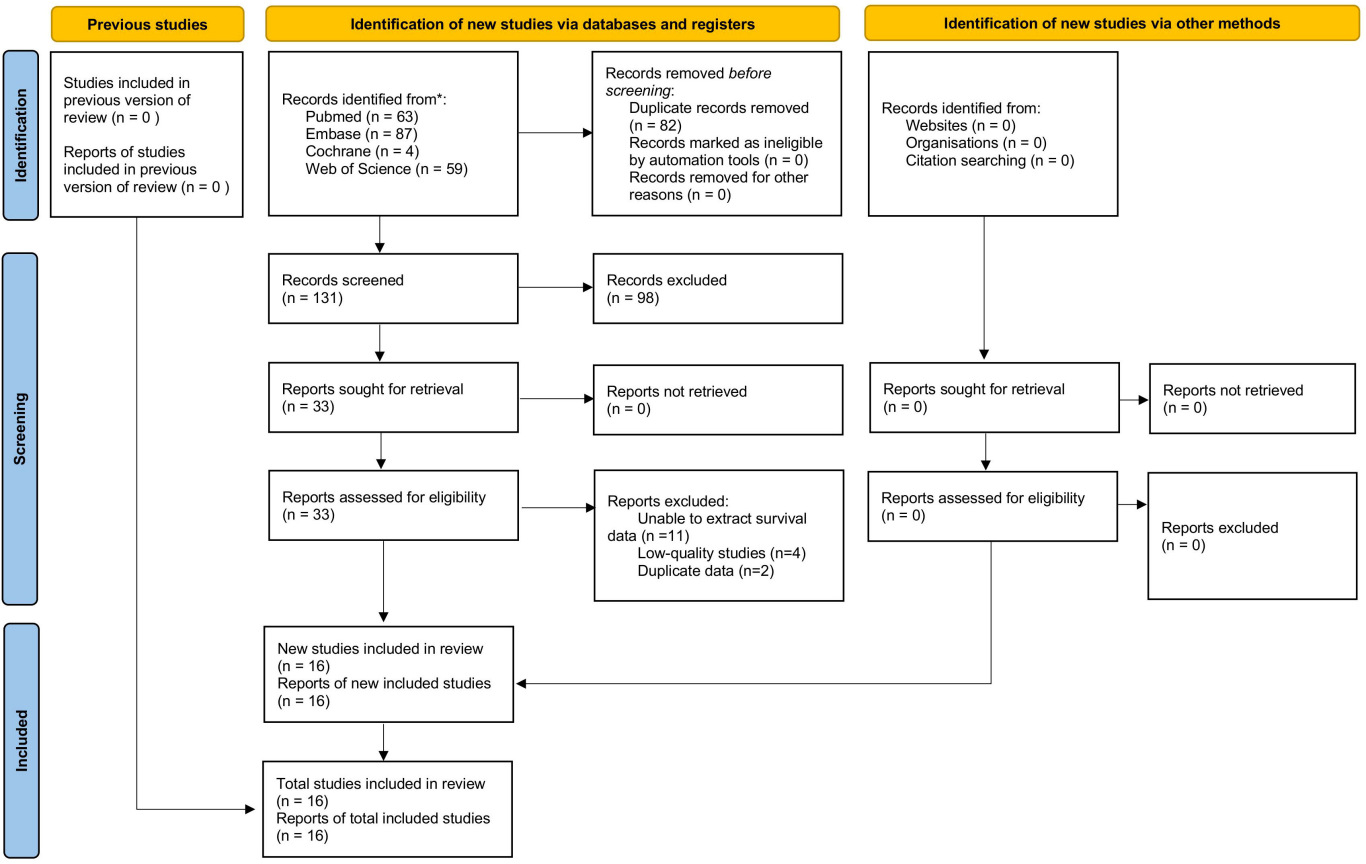
Flowchart of the systematic search and selection process.

**Figure 1** shows the flowchart of the literature retrieval and selection process. A total of 213 related studies in PubMed (n = 63), Embase (n = 87), Web of Science (n = 59), Cochrane (n = 4) were identified via systematically literature search. After removing duplicate studies, a total of 131 titles and abstracts were evaluated. Eventually, 16 cohort studies including 2352 patients were included for meta-analysis (18, 19, 25–38). **Table 1** presents the characteristics and quality evaluation of each eligible cohort study. Detailed NOS score results are shown in **Supplementary Table S3**.

**Table 1:** Characteristic and quality assessment of included studies.

Study	Country	Study design	Population	No. of patients	Gender		Mean/median age	TNM stage	SII cut-off	NOS score
					Male	Female				
Demirci 2025 (19)	Turkey	Retrospective	The elderly patient population diagnosed with non-muscle ınvasive bladder cancer	173	150	23	75.6	Ta-T1	656.03	7
Ding 2023 (30)	China	Retrospective	Patients with non-muscle-invasive bladder cancer receiving TURBT	282	243	39	66	Ta-T1	505	9
Grossmann 2022 (25)	Turkey	Retrospective	Muscle invasive bladder cancer who underwent radical cystectomy	191	NA	NA	62.1	T2-T4	843	7
Katayama 2021 (32)	US and Europe	Retrospective	Patients with NMIBC who underwent transurethral resection of bladder (TURB)	1117	855	262	67	Ta-T1	580	8
Ke 2021 (28)	China	Retrospective	Patients with non-muscle invasive bladder cancer	184	111	73	61.88	Ta-T1	439.8333	7
Kayar 2025 (38)	Turkey	Retrospective	Nonmetastatic Muscle-Invasive Bladder Cancer	115	101	14	65	T2-T4	692.5	7
Deng-Xiong 2023 (35)	China	Retrospective	Non-muscle-invasive Bladder Cancer Patients Receiving Intravesical Bacillus Calmette -Guerin	197	170	27	64.17	Ta-T1	557	9
Liu 2022 (29)	China	Retrospective	Patients with non-muscle invasive bladder cancer	183	143	40	62.37	Ta-T1	514.47322	8
Russo 2023 (26)	Italy	Retrospective	Patients with non-metastatic bladder cancer and no other histological variants who underwent radical cystectomy	193	154	39	NA	T0-T4	640.27	7
Salari 2024 (18)	Iran	Retrospective	Urothelial Bladder Cancer Following Radical Cystectomy	187	179	8	60.8	T0-T4	410.66	7
Yi 2023 (33)	China	Retrospective	Primary bladder cancer	496	384	112	NA	Ta-T1	525	7
Yilmaz 2023 (37)	Turkey	Retrospective	Patients with bladder cancer who underwent radical cystectomy	241	205	36	65	T1-T4	1228	7
Zhang 2019 (36)	China	Retrospective	Bladder cancer patients after radical cystectomy	209	182	27	NA	Tis-T4	507	9
Zhang 2022 (31)	China	Retrospective	Patients with bladder cancer after radical cystectomy	725	621	104	65	T0-T4	554.23	8
Zhang 2023 (2)	China	Retrospective	Patients with Muscle-Invasive Bladder Cancer	94	84	10	NA	NA	863.62	8
Zhao 2021 (27)	China	Retrospective	Non- muscular invasive bladder cancer patients	216	164	52	NA	Ta-T1	276.685	8

### OS

**Figure 2:**
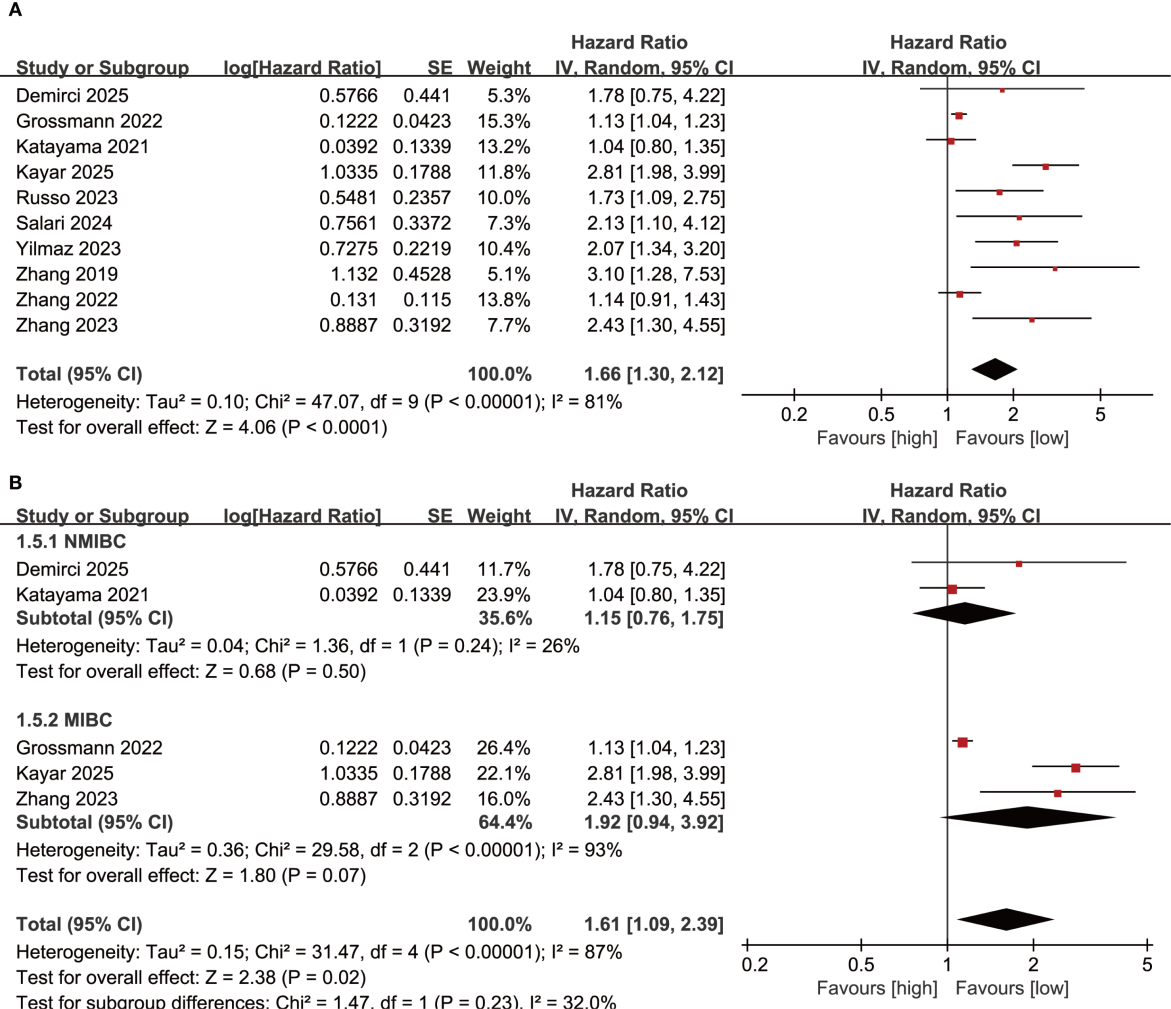
**(A)** Forest plots of OS; **(B)** Subgroup analysis of OS based on bladder cancer pathological type.

Results of OS were synthesized from 10 cohort studies, and meta-analysis revealed a significantly shorter OS in the group with high SII compared with the group with low SII (HR: 1.66; 95% CI: 1.30, 2.12; *P*<0.0001). A significant heterogeneity was observed (*I* ^2^ = 81%, *P*<0.00001) (**Figure 2A**). Subgroup analysis based on bladder cancer pathological type showed that SII had no effective prognostic value for both NMIBC (HR: 1.15; 95% CI: 0.76, 1,75; *P*=0.50) and MIBC (HR: 1.92; 95% CI: 0.94, 3.92; *P*=0.07) (**Figure 2B**). Furthermore, heterogeneity analysis revealed a significant decrease in heterogeneity in the NMIBC subgroup (*I* ^2^ = 26%) (**Figure 2B**).

### RFS

**Figure 3:**
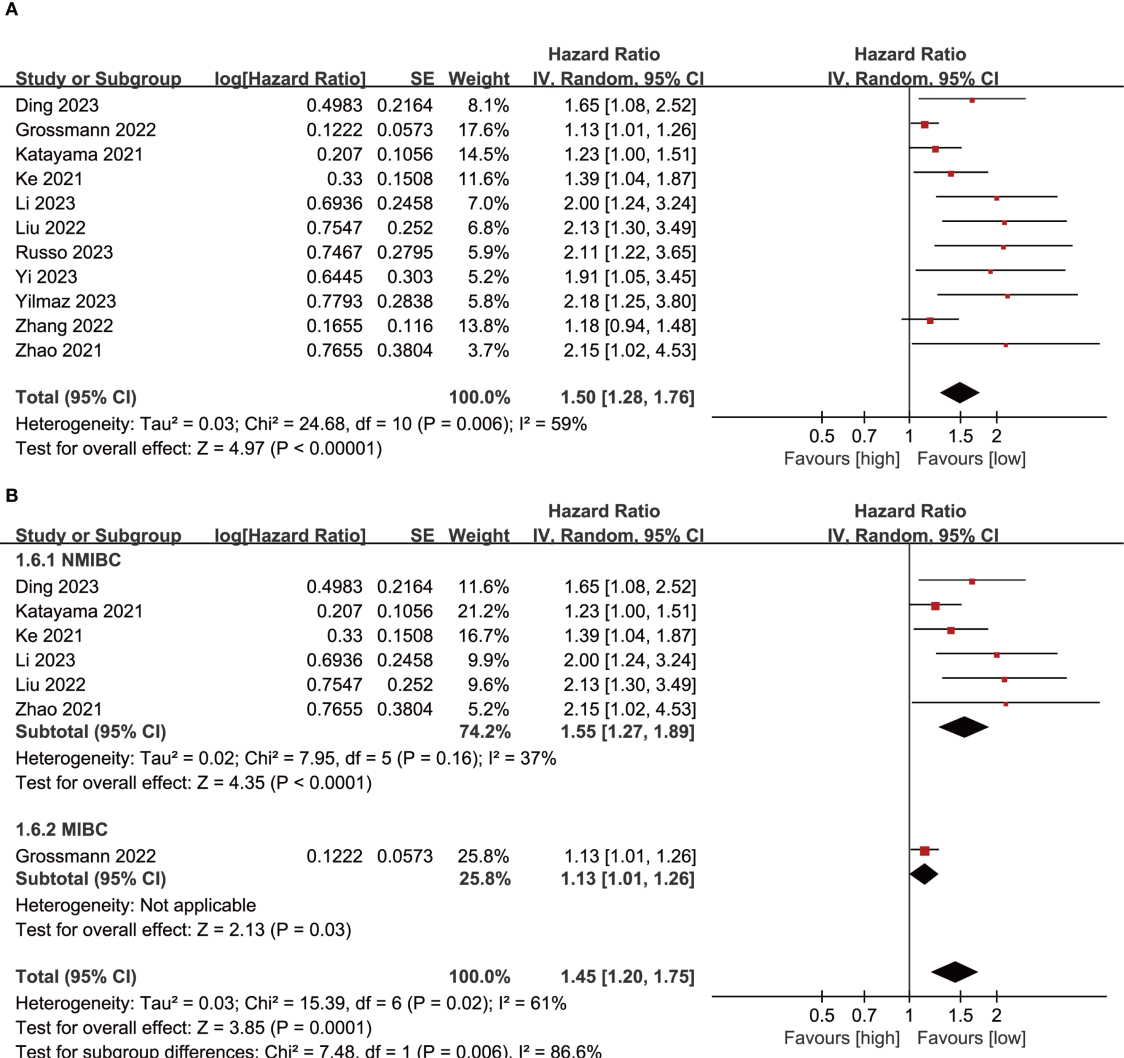
**(A)** Forest plots of RFS; **(B)** Subgroup analysis of RFS based on bladder cancer pathological type.

Results of RFS were synthesized from 11 cohort studies, and meta-analysis revealed a significantly shorter RFS in the group with high SII compared with the group with low SII (HR: 1.50; 95% CI: 1.28, 1.76; *P*<0.00001). A significant heterogeneity was observed (*I* ^2^ = 59%, *P*=0.006) (**Figure 3A**). Subgroup analysis based on bladder cancer pathological type revealed that SII had significant prognostic value for both NMIBC (HR: 1.55; 95% CI: 1.27, 1.89; *P*<0.0001) and MIBC (HR: 1.13; 95% CI: 1.01, 1.26; *P*=0.03). Furthermore, heterogeneity analysis revealed a significant decrease in heterogeneity in the NMIBC subgroup (*I* ^2^ = 37%) (**Figure 3B**).

### PFS

**Figure 4:**
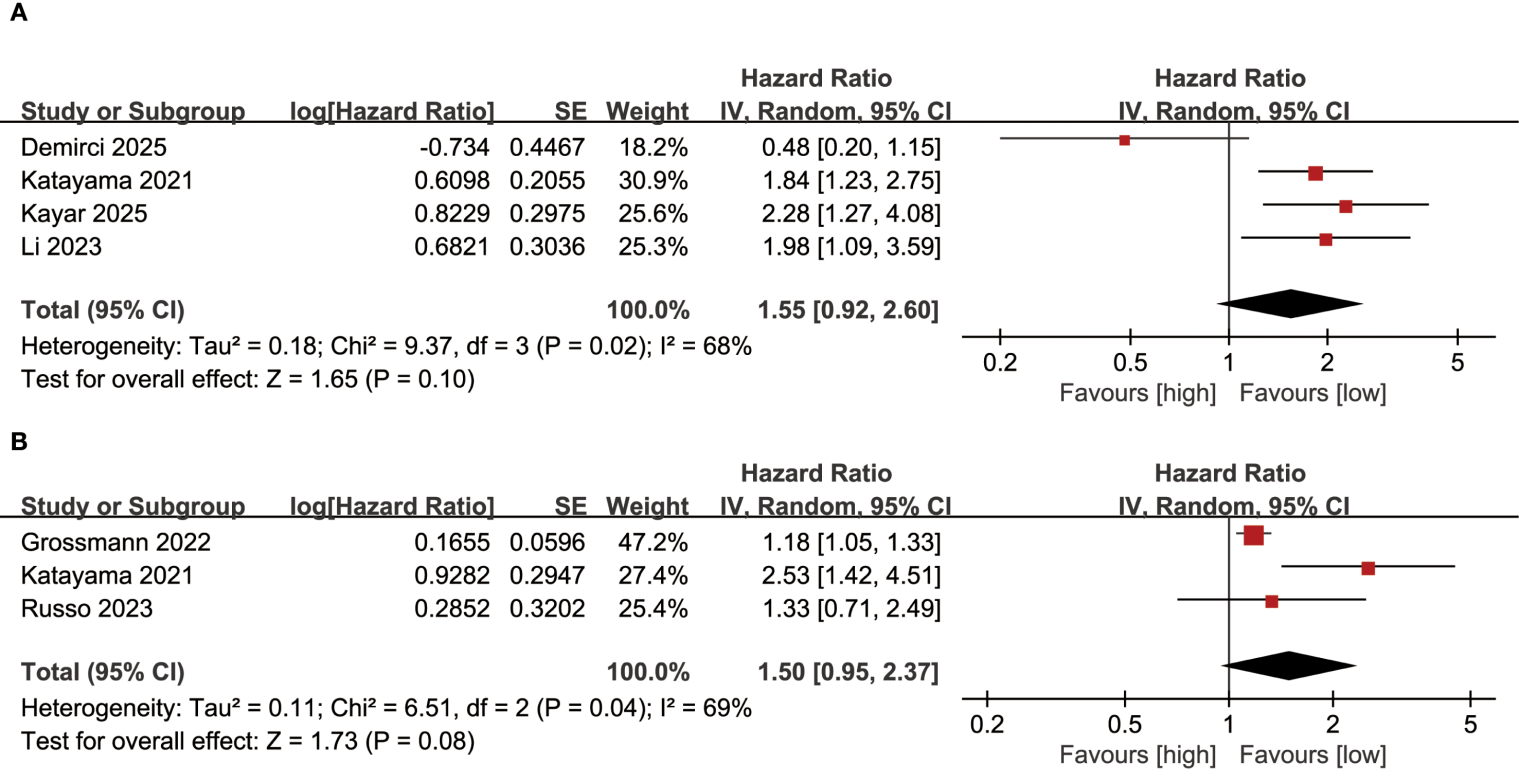
Forest plots of **(A)** PFS and **(B)** CSS.

Results of PFS were synthesized from 4 cohort studies, and meta-analysis found no significant correlation between SII and PFS in bladder cancer patients (HR: 1.55; 95% CI: 0.92, 2.60; *P*=0.10). A significant heterogeneity was observed (*I* ^2^ = 68%, *P*=0.02) (**Figure 4A**).

### CSS

Results of CSS were synthesized from 3 cohort studies, meta-analysis found no significant correlation between SII and CSS in bladder cancer patients (HR: 1.50; 95% CI: 0.95, 2.37; *P*=0.08). A significant heterogeneity was observed (*I* ^2^=69%, *P*=0.04) (**Figure 4B**).

### Publication bias and sensitivity analysis

**Figure 5:**
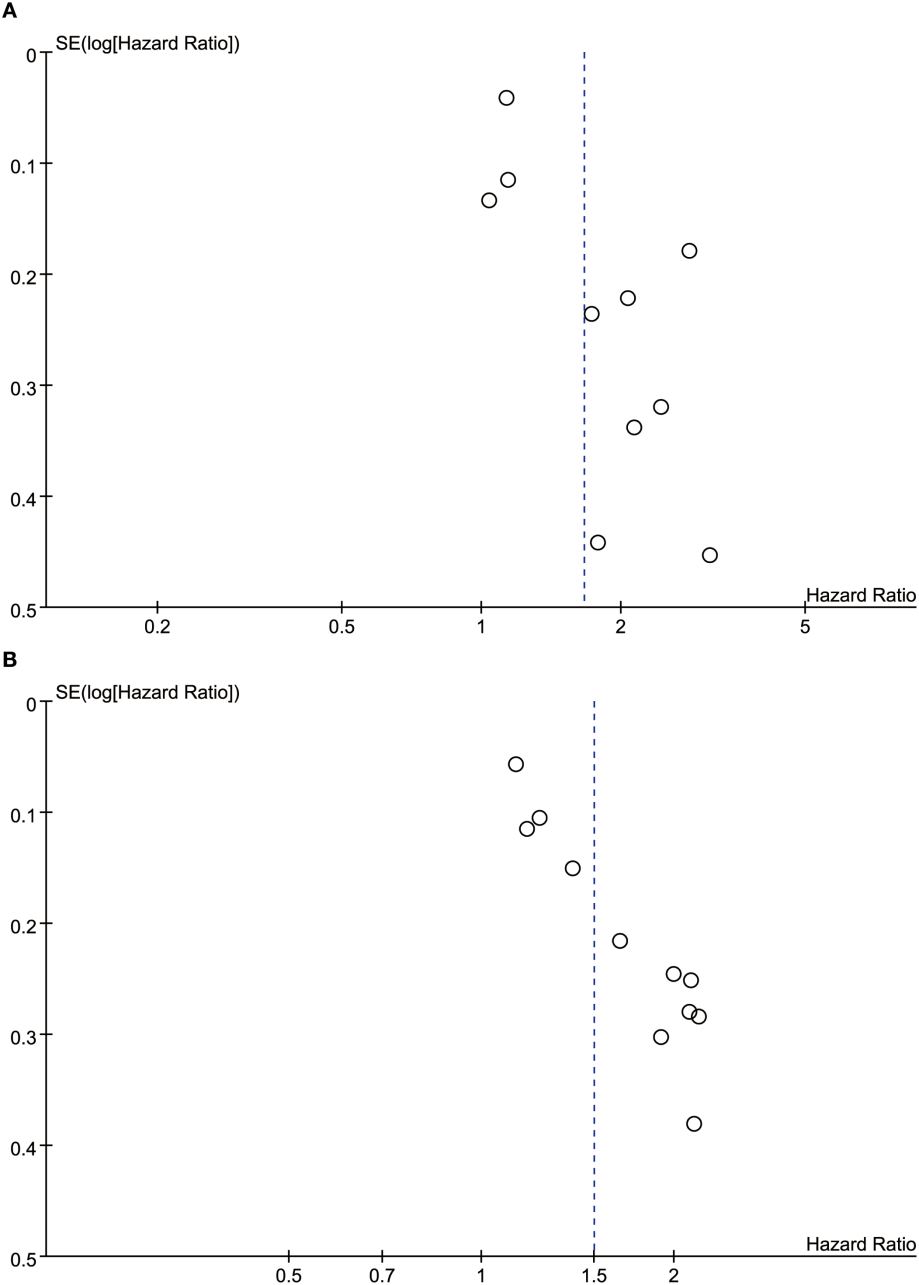
Funnel plots of **(A)** OS and **(B)** RFS.

**Figure 6:**
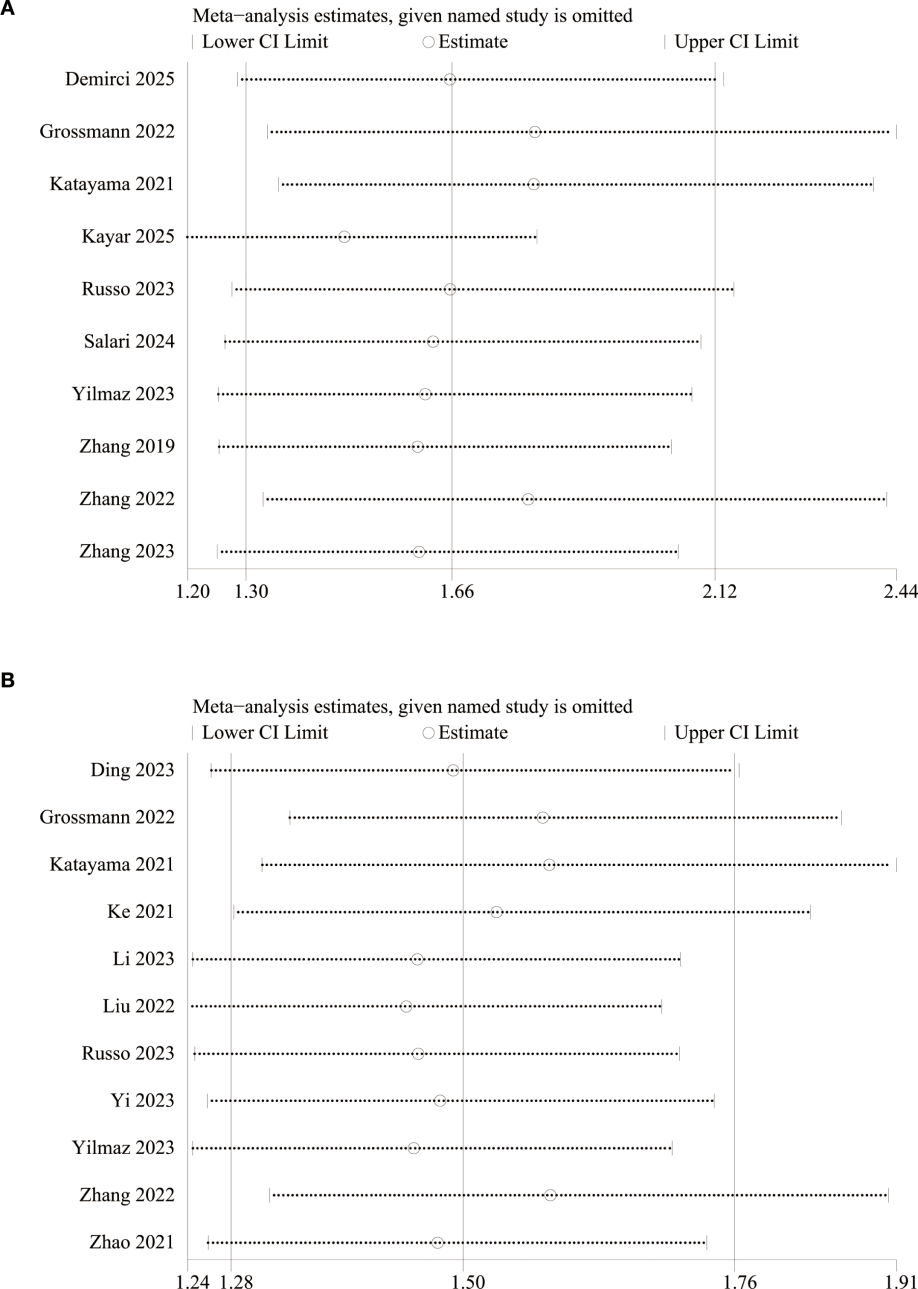
Sensitivity analysis of **(A)** OS and **(B)** RFS.

We assessed the potential publication bias through funnel plots and Egger’s regression tests for OS and RFS. Funnel plots and Egger’s test both showed significant publication bias in OS (**Figure 5A**, *P*=0.011) and RFS (**Figure 5B**, *P*=0.001). In addition, we performed sensitivity analysis for the results of OS and RFS to assess the effect of each cohort study on the total HR via excluding eligible cohort studies one by one. Sensitivity analysis found that the new total HR kept stable after removing of each cohort study for OS (**Figure 6A**) and RFS (**Figure 6B**).

## Discussion

This study, conducted through a systematic meta-analysis of 16 cohort studies (enrolling a total of 2,352 bladder cancer patients) through August 2025, comprehensively evaluated the association between SII and bladder cancer prognosis. The results showed that elevated SII was significantly associated with worse OS, with a pooled HR of 1.66 (95% CI: 1.30–2.12, P < 0.0001). High SII levels also predicted shorter RFS, with a pooled HR of 1.50 (95% CI: 1.28–1.76, P < 0.00001). Subgroup analyses revealed that SII had a significant predictive value for recurrence risk in patients with NMIBC (HR = 1.55). This association was also observed in patients with MIBC, but to a lesser extent (HR = 1.13). However, the predictive value of SII for PFS and CSS did not reach statistical significance. It should be pointed out that this study has obvious heterogeneity and potential publication bias, and all evidence comes from retrospective studies. These factors need to be fully considered when interpreting the results.

This study, through a meta-analysis, confirmed that elevated SII is significantly associated with poor prognosis in bladder cancer patients. This finding has profound implications for tumor immunobiology. As a systemic inflammatory marker, SII integrates information from three immune cell types: neutrophils, platelets, and lymphocytes (39). Its elevation reflects the formation of a pro-tumor immune microenvironment. Neutrophils promote angiogenesis and extracellular matrix degradation by secreting factors such as interleukin-8 (IL-8) and matrix metalloproteinase-9 (MMP-9), accelerating tumor invasion and metastasis (40). Platelets, through the release of transforming growth factor-β (TGF-β) and platelet-derived growth factor (PDGF), induce epithelial-mesenchymal transition (EMT) and inhibit cytotoxic T lymphocyte function (41). Depletion of lymphocytes, particularly CD8+ T cells, leads to impaired immune surveillance (42). This triple imbalance in the bladder cancer microenvironment contributes to an immunosuppressive state, promoting tumor cell evasion of immune clearance. This study observed a strong association between elevated SII and significantly shortened OS (HR=1.66, P<0.0001) and RFS (HR=1.50, P<0.00001), providing clinical evidence for the “inflammation-tumor progression” theory.

This study found significant differences in the prognostic value of SII for different clinical endpoints and bladder cancer subtypes, a stratification effect with important clinical implications. Regarding overall survival, a high SII level was associated with a 66% increased risk of death. However, subgroup analysis revealed that this association did not reach statistical significance in NMIBC (HR=1.15, P=0.50) or MIBC (HR=1.92, P=0.07), reflecting the confounding of multiple factors in OS prognosis. In contrast, SII demonstrated significant predictive value for recurrence risk in NMIBC (HR=1.55, P < 0.0001) but was less effective in MIBC (HR=1.13, P=0.03). This discrepancy may be due to the distinct biological nature of the two bladder cancer types: NMIBC recurrence is often driven by a dysregulated *in situ* immune microenvironment, while MIBC progression is more likely to involve systemic metastatic mechanisms (43, 44). Despite pooled HRs of 1.55 and 1.50 for PFS and CSS, respectively, no significant associations were established due to insufficient inclusion of studies (only four and three) and high heterogeneity (I² = 68%-69%). From a clinical perspective, the core value of the SII lies in its convenience: as a derivative of routine blood tests, it is low-cost and results are available within 24 hours, making it particularly suitable for primary care settings. Based on this evidence, a tiered approach is recommended for clinical practice: routine postoperative SII testing for NMIBC patients. When the SII exceeds the optimal cutoff, an intensive follow-up program (such as cystoscopy and urine cytology every three months) should be initiated; for MIBC patients, a comprehensive evaluation combining imaging and molecular markers is required (45). The value of dynamic SII monitoring is even more noteworthy: changes in SII before and after surgery may better reflect treatment response than single-measurement values. However, this study was limited by its retrospective design and was unable to analyze this dimension.

The high degree of heterogeneity across studies (pooled I² = 81% for OS and 59% for RFS) is a key point of contention in these results. Subgroup analyses provide key insights into the sources of heterogeneity. After stratification by pathology type, OS heterogeneity in the NMIBC subgroup was significantly reduced (I² decreased from 81% to 26%), suggesting that differences in study populations were the primary confounding factor. A thorough analysis of the primary study characteristics revealed three sources of heterogeneity: first, lack of standardization of SII testing, with significant variation in cutoff values ​​across studies and lack of standardized testing timing; second, variability in endpoint definitions, including RFS criteria (cytology-positive vs. pathologically confirmed recurrence vs. radiographic progression); and most importantly, confounding by treatment modalities: included studies included a variety of interventions, including TURBT, radical cystectomy, and intravenous BCG therapy, without adjustment for the impact of different treatments on inflammatory status. The unique performance of the MIBC subgroup is particularly noteworthy: although the pooled HR for OS reached 1.92, the wide confidence interval (0.94-3.92) and limited sample size (only three studies) significantly underpowered the statistical power. This reflects methodological flaws in MIBC research: large studies have focused on postoperative survival analysis but have not excluded patients receiving neoadjuvant chemotherapy. Furthermore, racial differences have not been adequately assessed: studies in Asian populations have generally reported higher SII effect sizes than those in European and American populations, possibly due to differences in genetic background. These confounding factors have led to conflicting conclusions in some studies: Ke et al. (28) reported significant predictive value of SII for NMIBC recurrence, while Zhang et al. (31) found negative results.

Limitations of this study profoundly impact the robustness and clinical applicability of the conclusions. First, all included studies in this meta-analysis were retrospective in design, subject to unavoidable selection bias. Most studies included only single-center samples and excluded patients with comorbid autoimmune diseases or infections, resulting in insufficient representation of the real world. Furthermore, sample size imbalance was significant. Although the RFS analysis included 11 studies, approximately 60% of the samples were concentrated in four large studies, while the CSS analysis included only three studies, resulting in significant statistical power deficiency. Second, the lack of standardization in SII measurement poses a significant challenge. Blood specimen processing time (from collection to detection) affects neutrophil activity, differences in detection systems between laboratories lead to high counting errors, and the influence of circadian rhythms on inflammatory markers was not considered. In addition, possible racial or regional differences in the prognostic value of SII need to be carefully considered. For example, studies on Asian populations included in this analysis often show higher effect sizes than those in European and American studies. This may be due to differences in genetic background, lifestyle, or medical system. Therefore, we recommend that future studies conduct stratified analyses by population to verify the universality of the conclusions of this study. Furthermore, key prognostic covariates were inadequately controlled: only five studies adjusted for TNM stage, and only two adjusted for molecular subtypes. Immunotherapy-related markers such as PD-L1 expression were not included in the analysis. At the same time, the lack of dynamic monitoring of treatment response is also a problem that cannot be ignored. All studies used baseline or postoperative SII values ​​and failed to evaluate the impact of SII changes during neoadjuvant chemotherapy or immunotherapy on prognosis. Finally, the problem of publication bias is even more serious. The Egger test showed that there was a significant publication bias in both OS (P=0.011) and RFS (P=0.001), and the asymmetry of the funnel plot suggested that studies with negative results may not have been published. These limitations together make it difficult for the current level of evidence to support SII as an independent prognostic indicator in clinical guidelines, especially for the prognostic judgment of MIBC patients, which requires caution.

To overcome the current evidence bottleneck, the following targeted research is urgently needed. The primary task is to establish a globally unified standard operating procedure (SOP) for SII testing. Core components include: clearly defined testing timing; standardized anticoagulation protocols; standardized specimen processing procedures; and a cross-platform calibration system. Regarding cohort design, a multicenter, prospective registry of inflammatory markers in bladder cancer should be initiated, with predefined key subgroups: stratified by pathological type (NMIBC vs. MIBC), treatment modality (surgery vs. chemoradiotherapy vs. immunotherapy), and molecular subtype (basal vs. luminal), using standardized endpoints. Furthermore, mechanistic insights are needed. Future studies could elucidate the spatial relationship between SII components and the tumor immune microenvironment using multicolor flow cytometry, establish a characteristic immunogenomic profile of patients with high SII using single-cell sequencing, and conduct organoid co-culture experiments to validate the effects of inflammatory mediators on bladder cancer cell stemness. Of greatest translational value are interventional studies, which would randomly assign patients with NMIBC and high SII to standard BCG instillation versus combined anti-inflammatory therapy for recurrence prevention, while also monitoring the correlation between dynamic changes in SII during treatment and remodeling of the tumor immune microenvironment.

## Conclusion

High SII was associated with shorter OS and RFS in patients with bladder cancer. Due to the simplicity and low cost of routine blood test, SII can be widely used to evaluate prognosis and construct risk prediction model for bladder cancer. Given the limitations of retrospective studies, population selection bias, and potential heterogeneity, more large-scale, multi-center, prospective clinical studies are needed to further validate the relationship between SII and the prognosis of patients with bladder cancer.

## Data Availability

The original contributions presented in the study are included in the article/**Supplementary Material**. Further inquiries can be directed to the corresponding author.
