# Supplementary material for: Prognostic value of the systemic immune-inflammation index in bladder cancer: an update evidence-based analysis
Source: Front Oncol. 2025 Oct 24;15:1707657. doi: 10.3389/fonc.2025.1707657 (PMC12591970; doi:10.3389/fonc.2025.1707657)
Supplement: Supplementary file 2 [file DataSheet2.docx]

Pubmed-63

((systemic immune-inflammation index) OR (SII)) AND (("Urinary Bladder Neoplasms"[Mesh]) OR ((((((((Urinary Bladder Neoplasm) OR (Bladder Neoplasm)) OR (Bladder Tumor)) OR (Urinary Bladder Cancer)) OR (Bladder Cancer)) OR (Cancer of Bladder)) OR (Malignant Tumor of Urinary Bladder)) OR (Bladder Carcinoma)))

Embase-87

((systemic immune-inflammation index or SII) and (Urinary Bladder Neoplasms or (Urinary Bladder Neoplasm or Bladder Neoplasm or Bladder Tumor or Urinary Bladder Cancer or Bladder Cancer or Cancer of Bladder or Malignant Tumor of Urinary Bladder or Bladder Carcinoma))).af.

Cochrane-4

((systemic immune-inflammation index or SII) and (Urinary Bladder Neoplasms or (Urinary Bladder Neoplasm or Bladder Neoplasm or Bladder Tumor or Urinary Bladder Cancer or Bladder Cancer or Cancer of Bladder or Malignant Tumor of Urinary Bladder or Bladder Carcinoma))).af.

WOS-59

((systemic immune-inflammation index) OR (SII)) AND ((Urinary Bladder Neoplasms) OR ((((((((Urinary Bladder Neoplasm) OR (Bladder Neoplasm)) OR (Bladder Tumor)) OR (Urinary Bladder Cancer)) OR (Bladder Cancer)) OR (Cancer of Bladder)) OR (Malignant Tumor of Urinary Bladder)) OR (Bladder Carcinoma))) (Topic)
