# Supplementary material for: Prognostic value of the systemic immune-inflammation index in bladder cancer: an update evidence-based analysis
Source: Front Oncol. 2025 Oct 24;15:1707657. doi: 10.3389/fonc.2025.1707657 (PMC12591970; doi:10.3389/fonc.2025.1707657)
Supplement: Supplementary file 3 [file DataSheet3.docx]

| Supplementary Table S3. Quality evaluation of the eligible studies with Newcastle–Ottawa scale. | | | | | | | | | |
| --- | --- | --- | --- | --- | --- | --- | --- | --- | --- |
| Study | Selection | | | | Comparability | | Outcome | | |
|  | Representative-ness | Selection of  non-exposed | Ascertainment  of exposure | Outcome not present at start | Comparability on most important factors | Comparability on other risk factors | Assessment of outcome | Long enough follow-up (median≥1 year) | Adequacy  (completeness) of follow-up |
| Demirci 2025 | * | * | * | * | - | - | * | * | * |
| Ding 2023 | * | * | * | * | * | * | * | * | * |
| Grossmann 2022 | * | * | * | * | - | - | * | * | * |
| Katayama 2021 | * | * | * | * | * | - | * | * | * |
| Ke 2021 | * | * | * | * | - | - | * | * | * |
| Kayar 2025 | * | * | * | * | - | - | * | * | * |
| Li 2023 | * | * | * | * | * | * | * | * | * |
| Liu 2022 | * | * | * | * | * | - | * | * | * |
| Russo 2023 | * | * | * | * | - | - | * | * | * |
| Salari 2024 | * | * | * | * | - | - | * | * | * |
| Yi 2023 | * | * | * | * | - | - | * | * | * |
| Yilmaz 2023 | * | * | * | * | - | - | * | * | * |
| Zhang 2019 | * | * | * | * | * | * | * | * | * |
| Zhang 2022 | * | * | * | * | * | - | * | * | * |
| Zhang 2023 | * | * | * | * | * | - | * | * | * |
| Zhao 2021 | * | * | * | * | * | - | * | * | * |
| *indicates criterion met; - indicates significant of criterion not met. | | | | | | | | | |
